# Effect of low-frequency assisted ultrasonic on cryopreservation of L-02 hepatocyte cells

**DOI:** 10.3389/fcell.2025.1571198

**Published:** 2025-04-28

**Authors:** Weijie Li, Xi Yang, Wenyan Bi, Liyong Song, Baolin Liu

**Affiliations:** ^1^ Institute of Biothermal Science and Technology, University of Shanghai for Science and Technology, Shanghai, China; ^2^ College of Chemistry and Chemical Engineering, Henan Polytechnic University, Jiaozuo, Henan, China; ^3^ Co-Innovation Center for Energy Therapy of Tumors, Shanghai, China; ^4^ Shanghai Technical Service Platform for Cryopreservation of Biological Resources, Shanghai, China

**Keywords:** ultrasonic ice seeding, ultrasonic intensity, hepatocyte, cryopreservation, hepatic function

## Abstract

Developing bioartificial liver and hepatocyte transplantation technology causes increasing hepatocyte cell demand. Effective long-term hepatocyte cell preservation methods are necessary to promote. Progressive cooling is a key preservation technology for cell banks. However, the cell solution needs to be supercooled in a slow freezing process. The high degree of supercooling possibly induces uncontrollable intracellular ice formation. This work designs an ultrasonic ice-seeding system for L-02 hepatocyte cell preservation, reducing supercooling and improving cell survival rate. The effect of ultrasonic intensities on the hepatocyte’s survival rate was investigated and optimized. The results prove the calorimetric method can efficiently measure the ultrasonic intensity dissipated in the hepatocyte cell preservation solution. When the ultrasonic intensity is 0.0329 W/cm^2^ ∼ 0.4316 W/cm^2^, the hepatocyte survival rate is over 90%. There is no significant difference between experiment groups (p < 0.05) when the ultrasonic intensity is larger than 0.4316 W/cm^2^. The hepatocyte cell survival rate reduced significantly with the increase of ultrasonic intensity. The 7-day hepatic function indicator experiment results indicate that the ultrasonic ice seeding has the weakest impact on hepatocyte cells in the four groups. The secretion of urea, albumin and glucose proved that ultrasonic ice seeding technology does not affect cell secretion and has an enormous advantage in cryopreservation. It can be widely applied to cell freezing fields.

## 1 Introduction

Low temperature is beneficial for the cell’s long-term preservation. The cooling rate has an enormous impact on the cell’s survival. In the long-term hepatocyte preservation process, cells were slowly cooled down to below the freezing point of the cryoprotection solution. Crystal nuclei were then placed in the cryoprotectant to accelerate the nucleation of the cell solution. Finally, continue to cool the cells to below −80°C. Ice nucleates and grows in the extracellular space (Da Costa et al., 2024). However, uncontrolled spontaneous ice nucleation is stochastic. It is detrimental and lethal to cells (Baklanov and Kiselev, 2023; D'Adamo et al., 2023; Qin et al., 2024; Silva et al., 2024). The lower the ice nucleation temperature, the more ice embryo nucleation (Silva et al., 2024). At a low subzero temperature like −10°C or below, the fine ice crystals formed outside cells easily pierce the cell membrane, causing physical damage and inducing intracellular water to form fine ice crystals. Supercooling conditions will lead to a high intracellular ice formation (IIF) tendency, which is lethal to cells (Baklanov and Kiselev, 2023; D'Adamo et al., 2023; Qin et al., 2024; Silva et al., 2024). In addition, sudden/rapid ice formation will cause the rapid osmosis of the extracellular solution around the growing ice crystals, inducing osmotic shock-type cell damage (OSD) (Silva et al., 2024).

In contrast, controlled ice nucleation at a high subzero temperature enables a reduced nucleation of ice embryos. They gradually grow into large ice crystals outside cells with further cooling, which leaves enough time for intracellular water to diffuse out of cells progressively and minimizes both the IIF and the OSD (Baklanov and Kiselev, 2023; D'Adamo et al., 2023; Qin et al., 2024; Silva et al., 2024). It is crucial for cryopreserving stress-sensitive cells like embryos. The impact degree of cryopreservation is possibly cell-type dependent (Silva et al., 2024; Vorobeva et al., 2023). Several methods control the ice nucleation number of samples during cryopreservation to reduce the damage (Vorobeva et al., 2023; Karasor et al., 2022). Early studies introduced ice crystals into an undercooled sample to manually “seed” ice (O'Kelly Boit, 2023). Later, to reduce the risk of sample contamination, pre-cooled probes, metal rods, or forceps were used to create cold spots from the outside wall of the container (e.g., cryovial), thereby providing localized deep supercooling (usually below −20°C) to induce ice nucleation in a sample that is above −10°C (Silva et al., 2024; Vorobeva et al., 2023; Karasor et al., 2022; O'Kelly Boit, 2023; Parihar et al., 2023). However, manual ice seeding is complex and lengthy to standardize because it often requires multiple trials to induce ice formation. Ice seeding can reduce cell supercooling, produce smaller ice crystals and reduce cell damage. At the same time, it can effectively avoid recrystallization during the rewarming process, improving cell survival (Ahmed et al., 2018; William and Acker, 2020; Mousazadehkasin et al., 2023; Najafi et al., 2023).

The traditional ice seeding method places the cell cryovial in an ice bath at −4°C to pre-cool to sub-zero temperature. Then, the cryovial wall was touched with tweezers or metal needles and pre-cooled in liquid nitrogen (Altmaier et al., 2022; Daily et al., 2020; Jiang et al., 2023; Lindow, 2023; Liu et al., 2021; Mutsenko et al., 2023; Yadegari et al., 2024; Zhang et al., 2024), creating the cryostat local overcooling to induce crystallization. However, these ice seeding methods cause uneven formation of ice crystals. The temperature near the position where tweezers or metal needles touched is lower, and the ice crystals formed are larger. It is easy to cause damage to cells and affect the survival rate and integrity during cell cryopreservation.

In addition, adding ice nucleation bacteria can also induce freezing (Zhang et al., 2024; Abir and Shin, 2024; Daily et al., 2023; Dunn et al., 2023; Tomas et al., 2023), as well as electrodes (Eskandari et al., 2024; Javitt et al., 2023; Mehanna and Abla, 2022; William et al., 2023; Zheng et al., 2018), shaking, vibration, ultrasound (Chen et al., 2024; Maharana et al., 2023; Zilei et al., 2023), etc. Many methods, including changing the cooling rate (Andres et al., 2023), the pressure (Juckers et al., 2024), etc., are also used to induce freezing nucleation. The ultrasound-induced nucleation research focuses on freezing food materials, such as freeze-concentration, freeze-drying of food (Midya and Bandyopadhyay, 2024), medicines, vaccines (Midya and Bandyopadhyay, 2024; Grover and Negi, 2023; Kazarin et al., 2023; Mokhova et al., 2023; Parale et al., 2024; Song et al., 2024), etc.

However, literature on ultrasound-assisted cryopreservation of cells remains limited. Existing research mainly investigates the effects of ultrasound on the permeation of cryoprotectants into cells, tissues, or organs. For instance, ultrasound treatment has been shown to enhance the penetration of cryoprotectants in fish embryos. Yet, it fails to achieve a sufficiently high intracellular cryoprotectant concentration for successful vitrification (Rahman et al., 2017; Bart and Kyaw, 2003). Moreover, the excessive permeation of cryoprotectants at high concentrations poses a risk of cytotoxicity. Thus, further exploration is required to elucidate the role of ultrasound in the cryopreservation of biological tissues and cells. This study focuses on the effect of ultrasound on ice nucleation during cryopreservation, aiming to determine whether appropriate ultrasound treatment can overcome the limitations of conventional cryopreservation methods and improve cell survival rates after freezing.

In studying ultrasonic ice seeding-assisted crystallization, many factors are usually used to describe sonochemical power, including ultrasonic power, meter power, input ultrasonic power, output ultrasonic power, and so on (Nogueira et al., 2023). Thus, comparing the sonochemical results reported by different laboratories isn’t easy. It is called the “reproducibility problem in sonochemistry” (Xing et al., 2021). The energy is a kind of mechanical energy that converts electrical energy into ultrasonic waves through the vibrator. The converting efficiency depends not only on the model of the instrument but also on the size of the vibrator, the specifications of the ultrasonic container, etc. Energy loss occurs when the sensor converts electrical energy into ultrasonic energy. The energy dissipated in the ultrasonic container is less than the electrical power energy. Therefore, electrical energy can not describe sound energy accurately. Although it is not necessary to measure the energy as mechanical energy converted from electrical energy in all cases, there are some representative standard methods to determine ultrasonic energy generated by a particular instrument. There are two main methods to measure the ultrasonic power dissipated in the sample solution. One is the chemical dosimeter method, the Weissler reaction (Rajamma et al., 2021). The ultrasonic power dissipated in the chemical reaction system is indirectly determined by measuring the change of certain chemical substances. For example, ultrasonic water treatment containing carbon tetrachloride will produce chlorine molecules, reacting quickly with iodine ions in the solution to release iodine molecules. The amount of iodine released by potassium iodide under ultrasound can determine the ultrasonic power (*P*_s_). Measuring the amount of HNO_3_ produced by NO_3_ in the water under ultrasonic waves can also determine Ps.

However, not all liquid sound-transmitting media have sonochemical reactions and chemical dosing methods are rarely used. The other is calorimetry (Hamdaoui and Alghyamah, 2023). The heat energy received by the sample is measured to express the ultrasonic power dissipated.

This method assumes that the released ultrasonic energy can be absorbed by the liquid sound-transmitting medium and turned into thermal energy. Many experts recommend calorimetry as an effective method to obtain the power dissipated in the sample (Orozco M. A. et al., 2021). The power level measured by the calorimetry method is proportional to the instruments’ electrical input power, and the ultrasonic ice seeding instrument with different volumes and different shapes of containers can obtain more consistent results. Based on the above discussion of the method of ultrasonic power dissipated in the sample solution, this article adopts calorimetry to measure the ultrasonic power and ultrasonic intensity dissipated in the cell solution of different concentrations.

## 2 Materials and methods

### 2.1 Materials

L-02 primary hepatocyte (purchased from FuHeng Biology, Shanghai), fetal bovine serum (Gemini, Shanghai), medium RPMI-1640 (Gibco, Thermo Fisher Scientific), trypsin (TBD, Tianjin), dimethyl sulfoxide Me_2_SO (Aladdin, Shanghai), PBS buffer (TBD, Tianjin), ethylene glycol (Macklin, Shanghai), optical microscope (Nikon, Japan), fluorescence microscope (Nikon, Japan), low-speed Benchtop centrifuge (Anke, Shanghai), CO_2_ cell incubator (BoXun, Shanghai), program cooling box (Thermo Fisher Scientific, America), ultra-low temperature refrigerator (Haier, Qingdao), low-temperature thermostat (Tianheng, Ningbo), intelligent multi-channel Temperature tester (Anbai, Changzhou), differential scanning calorimeter (Netzsh, Germany).

### 2.2 Construction of ultrasonic ice seeding system

**FIGURE 1:**
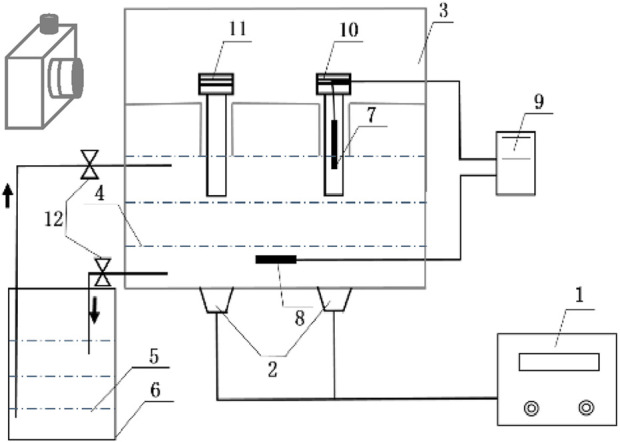
The schematic diagram of the ultrasonic ice seeding system. (1) Ultrasonic generator; (2) Ultrasonic vibrator; (3) Ultrasonic inner box; (4,5) Temperature control phase change liquid; (6) low-temperature thermostat; (7) First thermocouple; (8) Second thermocouple; (9) Temperature data acquisition module; (10) Control cryotube; (11) Hepatocyte cells cryotube; (12) Flow control valve.

Figure 1 is the schematic diagram of the ultrasonic ice seeding system. It consists of five parts: (I) The ultrasonic container model is designed according to the model of the program cooling box. The control cryotube and hepatocyte cryotubes can be placed in the sample tank. The bottom of the sample tank is connected with the phase change liquid, and the sample tank is placed in the ultrasonic inner box. The bottom material of the ultrasonic container is selected as an aluminium sheet. (II) Temperature detection system: It comprises the first thermocouple electrode, the second thermocouple electrode and the temperature data acquisition module. The first thermocouple electrode is placed in the control cryotube to detect the temperature of the cell solution in real-time. The thermocouple electrode is placed in the phase change liquid in the ultrasonic inner box for real-time detection of the temperature of the control phase change liquid. (III) Ultrasonic system: It consists of an ultrasonic vibrator and an ultrasonic generator (Shanghai Shengpu Ultrasonic Equipment Factory), in which the ultrasonic vibrator (diameter d = 45 mm) is uniformly bonded to the bottom of the ultrasonic container, and the ultrasonic wave propagates upward from the ultrasonic container to the tube. The ultrasonic frequency is 40 kHz, and the electric power range is 0∼150 W adjustable. (IV) Cooling circulation system: A low-temperature thermostat provides a cold source. Its temperature control range is - 25°C ± 0.1°C–25°C ± 0.1°C. Because ethylene glycol has the advantages of high boiling point, not easy to evaporate, not easy to catch fire, and good safety, this article uses 38.5% ethylene glycol as the freezing liquid, which can be cooled to −20°C without freezing. The ultrasonic inner box and the low-temperature thermostat are loaded with refrigerant, causing the closed circulation loop. The low-temperature thermostat has a refrigeration cycle system, and the temperature distribution in the ultrasonic container is uniform. (V) Photographic recording system: The CCD high-speed camera (Sony E3ISPM06300KPB, resolution 3,072 × 2,048 pixels, 59 frames/s) transmitted the video signal to the computer, and the effect of ultrasonic ice seeding is recorded in real-time.

### 2.3 Determination of specific heat capacity of hepatocyte solution

**FIGURE 2:**
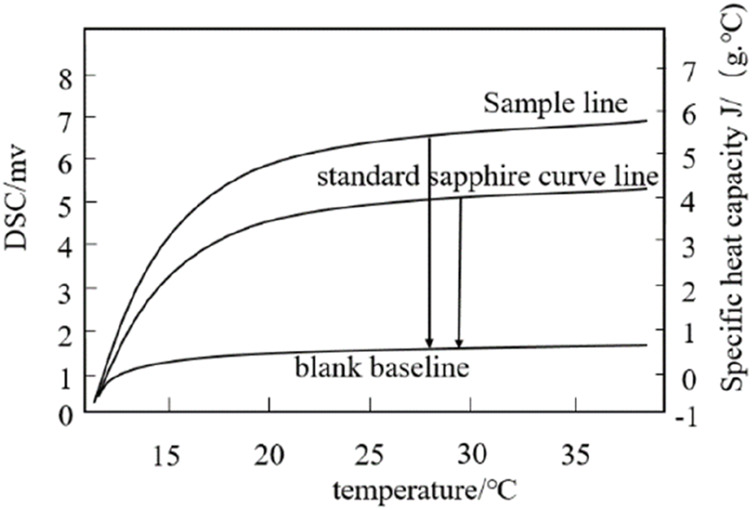
Determine the specific heat of the sample line, sapphire line and blank baseline.

The specific heat capacity of different Me_2_SO solutions was measured to control the ultrasonic power and ultrasonic intensity dissipated in the hepatocyte’s solution more accurately. The DSC three-wire method is used to determine the specific heat capacity of the cell solution (Orozco M. et al., 2021). The experimental principle is shown in Figure 2. First, place an empty crucible on the sample holder and reference holder in the furnace to measure a blank baseline. Second, the DSC curve of the known standard sample sapphire and the DSC curve of the cryopreservation solution were measured under the same conditions. During the test, the high nitrogen purge rate for cleaning was 20 mL/min for liquid nitrogen refrigeration, and the circulating water constant temperature system was set to 30°C. The control program is as follows: the initial temperature is 10°C, continuous time is 5 min, then the temperature is raised to 40°C at 5°C/min and kept at 40°C for 5 min. Before the cell solution test, press the sealer first, weigh the sample mass, and determine the cell solution sample line. The specific heat capacity of the cell solution at the required temperature is calculated by the Formula 1.

$$Cp\left(s\right)=\frac{Cp\left(st\right)\times Ds\times Wst}{Dst\times Ws}$$ (1)

Where *Cp(s)* (J/(g·°C)) is the specific heat capacity of the cell solution; *Cp(st)* (J/(g·°C)) is the specific heat capacity of the standard sample sapphire; *Ds* (mW) is the vertical displacement value between the sample line and the blank baseline of the DSC thermal curve at a specific temperature; *Dst* (mW) is the vertical displacement value between the standard sapphire curve line and the blank baseline of the DSC thermal curve at a particular temperature; *Ws* (mg)is the mass of the hepatocyte cells solution to be tested. *Wst* (mg) is the mass of the standard sapphire curve.

### 2.4 Determination of parameters of ultrasonic ice seeding system

The ultrasonic power range is 0∼150 W and can be adjusted. To reduce the workload, the actual ultrasonic power (*P*_*s*_) dissipated in the hepatocyte’s solution with different Me_2_SO concentrations was measured with the electric power of 30 W, 60 W, 75 W, 90 W, 105 W, 120 W, 135 W, and 150 W. This work uses calorimetry to measure the ultrasonic power (*P*_*s*_) dissipated in the hepatocyte solution. The calorimetry method assumes that the released ultrasonic energy can be absorbed by the liquid sound-transmitting medium and converted into heat energy. The calculation Formula 2 is as follows:

$${P_{s}=M\times C_{P}\times \left(dT/dt\right)}_{t=0}$$ (2)

Where *M*(g) is the mass of the liquid sound transmission medium, as the mass of the hepatocyte cells solution of different Me_2_SO; *Cp* (J/(g·°C) is the specific heat capacity *(dT/dt)*_*t*=0_ (°C/min) is the tangent slope of the liquid temperature changing with time at 0 s at the action of ultrasound. First, let the initial temperature of the phase change liquid in the ultrasonic ice seeding system and the cryopreservation tube equal to the room temperature (the temperature in this laboratory is 19°C). The ultrasonic time is 50 s due to the uneven ultrasonic intensity in different directions in the ultrasonic ice seeding instrument (the ultrasonic intensity right above the ultrasonic vibrator is much greater than in other places). The volume and depth of the phase change liquid significantly influence the ultrasonic intensity. Therefore, the experimental samples are all placed in the same position in the ultrasonic container (in this experiment, the samples are placed right above the ultrasonic vibrator). The depth of the phase change liquid is 100 cm higher than the bottom of the ultrasonic ice seeding instrument (the cryopreservation tube is immersed in the phase change liquid at the position of 1.5 cm). Each condition was repeated for three parallel experiments to obtain more accurate results. Sample cryotube and phase change solution were used only once in each experiment. The ultrasonic intensity (*I*_s_) can be calculated by Equation 3.

$$I_{s}=\frac{P_{s}}{A_{h}}$$ (3)

Where *I*_s_ is ultrasonic intensity; *P*_*s*_ (W) is the actual ultrasonic power dissipated in the sample; *P*_*s*_ is measured by calorimetry. *A*_*h*_ (cm^2^) is the cross-street area of the cryotube. The diameter of the cryopreservation tube is 10.20 mm, and the *A*_*h*_ is 0.8171 cm^2^.

### 2.5 Hepatocyte culture

L-02 hepatocyte cells were cultured with RPMI-1640 medium and 10% fetal bovine serum in an incubator at 37°C with an atmosphere of 5% CO_2_. Before subculture, observe the number of hepatocyte cells. When hepatocyte reaches 80%∼90%, discard the supernatant and add 1 mL PBS buffer. After washing with PBS twice, discard it. Then add 1 mL of trypsin. Put the culture flask in the incubator and enzymolyze the cells for 2 min. When hepatocyte cell morphology has become rounded under the optical microscope, gently tap the side of the T-25 culture flask. The hepatocyte cells can be separated from the surface of the wall. Add a 2 mL RPMI-1640 medium to terminate the digestion, and use a 1 mL pipette to suck the culture solution repeatedly to ensure they are detached from the bottle wall. Then, transfer it to the centrifuge tube and centrifuge it at 1,000 r/min for 4 min. Finally, the hepatocyte cells are resuspended in PBS solution for later use.

### 2.6 Cryopreservation of hepatocyte cells

**FIGURE 3:**
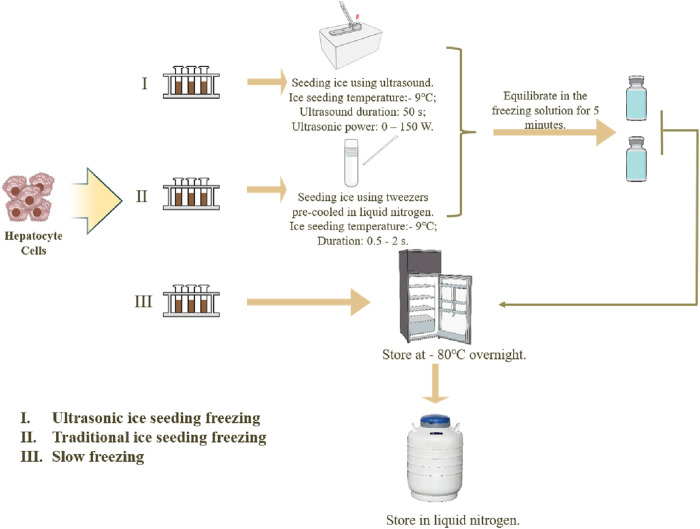
Three methods of hepatocyte cryopreservation.

Three methods for hepatocyte cell cryopreservation are used: ultrasonic ice seeding freezing, traditional ice seeding freezing, and slow freezing, as shown in Figure 3.

Each method has three parallel experiment groups. (I) The ultrasonic ice seeding freezing method: Prepare 38.5% (v/v) ethylene glycol in advance and lower the temperature to −20°C. After the cell solutions were resuspended, 1,500 µL was sampled into three 2 mL tubes. Solutions without hepatocyte cells were added into control tubes. Then, the control tube and three hepatocyte tubes were put in the ultrasonic ice seeding instrument. First, connect the ultrasonic container with the cryostat. 38.5% (v/v) glycol carrier refrigerant is injected into the ultrasonic inner box and the cryostat. Then, turn on the low-temperature constant tank refrigeration cycle system. When the second thermocouple detects that the temperature of the solution reaches the specified temperature, keep the temperature constant. When the first thermocouple detects that the temperature of the control cryopreservation tube reaches the specified ice planting temperature, turn on the ultrasonic generator. After the ice seeding, turn off the ultrasonic and equilibrate the cell cryopreservation tube in the freezing solution for 5 min to allow the ice crystals to grow. After the equilibration, transfer the cell cryopreservation tube to the cooling box and keep it at −80°C overnight. Then, transfer the cryopreservation tube to liquid nitrogen for 24 h. (II) The cryopreservation method for traditional ice-seeding freezing: The cell solution is first cooled to the ice-seeding temperature. Tweezers pre-cooled in liquid nitrogen are then used to make direct contact with the outer wall of the cryovial. The tweezers should precisely touch a single point on the cryovial wall for approximately 0.5–2 s to induce nucleation. The contact time should be minimized to prevent excessive sample cooling and ensure controlled ice crystal formation. Following nucleation, the cryovial is equilibrated in the freezing solution for 5 min to allow complete ice crystal growth. After equilibration, the cryovial is transferred to a cooling box and stored at −80°C overnight. Finally, the cryovial is transferred to liquid nitrogen for 24 h (III) The slow freezing method: directly transfer the cell cryopreservation tube to the cooling box and place it at −80°C refrigerator overnight. Then, transfer it to liquid nitrogen for deep cryopreservation the next day.

### 2.7 Hepatocyte cell number and survival rate determination

After centrifugation, the hepatocyte cells were resuspended with 1 mL of culture medium. Under an optical microscope, 10 µL of resuspension solution was pipetted into the groove on one side of the hemocytometer. To evaluate the survival rate of the hepatocyte cells after cryopreservation, 15 µL of the suspension solution was drawn into a 1.5 mL centrifuge tube by a 15 µL pipette. Since AOPI dye is more accurate than trypan blue dye, 15 µL of the AOPI dye was mixed in the same centrifuge tube and incubated at 4°C in the dark for 10∼20 min. After incubation, draw 15 µL of cell staining solution in the center of the slide, cover it with a cover glass and observe through a fluorescent microscope. The wavelength of the excitation light source was 488 nm, and the wavelength of receiving light was 520 nm. The preparation of the AOPI staining solution is as follows: 100 µL reagent C was diluted with 900 µL PBS buffer, 10 µL AO staining solution and 20 µL PI staining solution were added. After the staining, find the cells in the bright field and observe them in the dark room with a fluorescent microscope. Finally, Image Pro Plus 6.0 software was used to count the number of hepatocyte cells.

### 2.8 Hepatocyte morphology observation and hepatocyte function test

#### 2.8.1 Hepatocyte morphological observation

The morphology changes of different cryopreserved hepatocyte cells were recorded by ordinary optical microscope from the 1st to the 7th day to test the hepatocyte’s function.

##### 2.8.1.1 Measurement of urea production

The urea concentration in the culture flask was measured using urease Bourbon colorimetry. Urease hydrolyzed urea, producing ammonia and carbon dioxide. Then, the ammonium ions react with phenol to produce blue indoxyl. The amount of indoxyl produced is directly proportional to the urea content.

Prepared a 5 mmol/L standard solution (standard urea solution (100 mmol/L): ddH_2_O = 1:19) and urease solution (urease solution: urea diluent = 1:99). Then added 10 µL ddH_2_O, standard urea solution (5 mmol/L) and the sample (culture flask supernatant) into a blank tube, standard tube and measuring tube, separately. Added 200 µL urease solution to each tube and kept in a 37°C water bath for 15 min. Then, 1 mL of phenol coloring solution and 1 mL urea assay buffer were added to each tube and kept in a water bath at 37°C for 20 min. The absorbance of solutions in each tube was measured at 560 nm by a microplate reader.

##### 2.8.1.2 Measurement of albumin synthesized

Bromocresol green has a high affinity for albumin. Albumin molecules with positive charges react with bromocresol green with negative charges, forming a blue-green complex with strong absorption at 628 nm. The absorbance of the complex is directly proportional to albumin. Thus, bromocresol green colorimetry was used to measure albumin content here.

A 40 mg/mL albumin standard solution was prepared and stored at 20°C. 10 μL albumin standard preparation solution, albumin standard solution (40 mg/mL) and sample (culture flask supernatant) were added to the blank, standard, and measurement tubes separately. Then, 2 mL bromocresol green was added to each tube and kept at room temperature for (30 ± 3) s. A microplate reader was used to measure the absorbance at 628 nm.

##### 2.8.1.3 Measurement of glucose content

Glucose reduces copper ions into cuprous oxide precipitation in a heated alkaline environment. Cuprous oxide reduces phosphomolybdic acid to molybdenum blue. The color depth is proportional to the glucose content. Thus, the Folin-Wu microplate method was used to detect the glucose content.

First, add 300 µL distilled water to the blank tube, 200 µL distilled water and 100 µL Glucose standard (10 mg/mL) to the standard tube, 200 µL distilled water and 100 µL supernatant to the test tube separately. Then, 100 µL Folin alkaline copper solution and 100 µL Wu phosphomolybdic acid solution were added to three tubes separately. After mixing well, boil three tubes in a water bath, take it out, and cool it down in cold water without shaking. A microplate reader was used to measure the absorbance of solutions in each tube at 420 nm.

### 2.9 Data analysis

The fluorescence images were analyzed using Image-Pro Plus 6.0 software. The data was processed by IBM SPSS Statistics 22.0 software. Each sample was repeated three times, representing all data as mean ± standard deviation. p < 0.05 was used as the criterion of significant difference, which was statistically significant.

## 3 Results and discussion

### 3.1 The specific heat capacity of different concentrations of Me_2_SO cell solution

According to the calculation Formula 2, the specific heat capacity of the cell solutions is obtained (Table 1). There is no significant difference in the specific heat capacity between 0%∼10% Me_2_SO. For the convenience of subsequent calculations, the average specific heat capacity of the four different concentrations solution of Me_2_SO is taken as the specific heat capacity of the cell solution. Therefore, the specific heat capacity of the hepatocyte’s solution is 3.997 J/(g.°C).

**TABLE 1:** Specific heat capacity of Me_2_SO (25°C).

Concentration of Me_2_SO (v/v) %	Specific heat capacity (J/(g.°C))	Mean ± standard
0	4.008 4.098 4.138	4.081 ± 0.07^a^
3	4.146 4.004 4.004	4.051 ± 0.08 ^ab^
5	3.902 3.969 3.978	3.950 ± 0.04 ^ab^
10	3.940 3.889 3.866	3.905 ± 0.03^b^

Note: Values with different superscripts (a, b) in the same column are significantly different (p < 0.05).

### 3.2 The ultrasonic power (*P*_s_) and intensity (*I*_s_) dissipated in the sample

**FIGURE 4:**
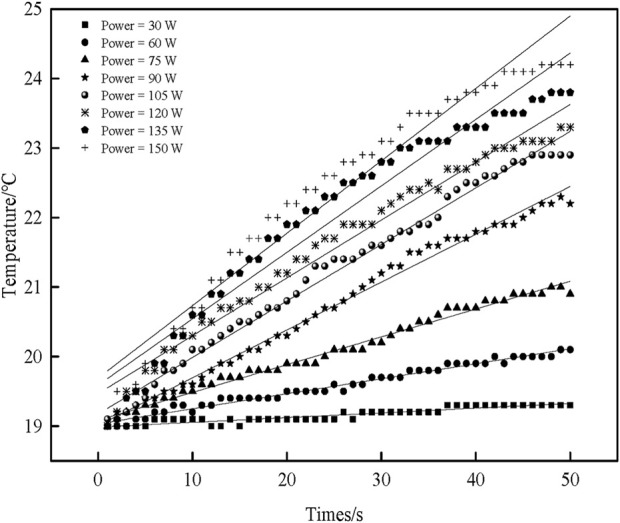
The effect of ultrasonic power on sample temperature over time.

Figure 4 shows the relationship between the sample temperature and the ultrasonic time under different ultrasonic powers.

There is a pronounced linear relationship. The sample temperature increases with the increase in ultrasonic time. Although a deviation from a strictly linear relationship is observed at higher ultrasonic power levels (120 W, 135 W, 150 W), this is a normal phenomenon caused by the rapid temperature rise, which leads to larger temperature differences and enhanced heat dissipation, ultimately reducing the internal heating rate. Nevertheless, the overall trend remains valid. Origin 8.0 is used to fit the linear equation, and the figure corresponds to the fitted linear equation shown in Table 2. Among them, the correction coefficient of the determination value is close to 1, indicating that the linear regression equation fits better. Therefore, the fitted linear regression equation can approximate the relationship between temperature and ultrasonic time.

**TABLE 2:** Linear regression equations and related parameters for the relationship between sample temperature and ultrasonic duration under different ultrasonic power.

Ultrasonic power (W)	Linear regression equation	Adj. R-R-square	Mass (g)	dT/dt_(t=0)_ (°C/s)	*P*_*s*_ (W)	*I*_*s*_ (W/cm^2^)
30	Y = 0.0068t + 18.99	0.8667	0.991	0.0068	0.0269	0.0329
60	Y = 0.0216t + 19.03	0.9788	0.986	0.0216	0.0851	0.1041
75	Y = 0.0400t + 19.08	0.9884	0.980	0.0436	0.1706	0.2088
90	Y = 0.0688t + 19.01	0.9919	0.984	0.0688	0.2706	0.3312
105	Y = 0.0813t + 19.17	0.9889	0.987	0.0813	0.3207	0.3925
120	Y = 0.0832t + 19.47	0.9770	0.977	0.0903	0.3527	0.4316
135	Y = 0.0956t + 19.59	0.9451	0.985	0.0956	0.3764	0.4607
150	Y = 0.1042t + 19.69	0.9503	0.986	0.1042	0.4107	0.5026

According to the fitted linear equation, the slope of the tangent of the temperature versus time can be obtained ((dT/dt) _t=0_). The mass of the sample under different ultrasonic powers is shown in Table 2. The specific heat capacity (*C*_*p*_) of the sample solution is 3.997 J/(g.°C). According to Formulas (2) and (3), the actual ultrasonic power (*P*_*s*_) and the ultrasonic intensity (*I*_*s*_) dissipated in the sample under different ultrasonic electric powers can be obtained, respectively.

### 3.3 Effect of ultrasonic ice seeding method on inducing nucleation

**FIGURE 5:**
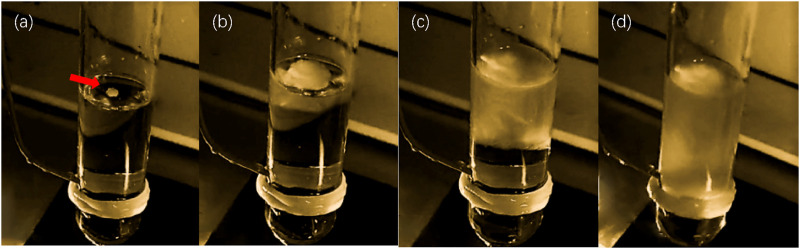
The effect of ultrasonic ice planting and nucleation. **(a)** Image of ice crystal growth after ultrasonic ice seeding for 1 s; **(b)** Image of ice crystal growth after ultrasonic ice seeding for 2 s; **(c)** Image of ice crystal growth after ultrasonic ice seeding for 4 s; **(d)** Image of ice crystal growth after ultrasonic ice seeding for 8 s.

To test the effect of the ultrasonic ice seeding method on inducing nucleation, the cryopreservation tube was replaced with a glass tube in this experiment. The glass tube has high transparency, which is convenient for observing the nucleation of ice crystals. In the experiment, start the ultrasonic ice seeding when the cell freezing solution in the glass test tube is pre-cooled to a temperature below zero. After the ultrasonic ice seeding, the glass test tube is removed from the ultrasonic container, and the growth of ice crystals is shown in Figure 5.

Notably, upon removal from the ultrasonic container, ice crystals preferentially nucleated at the air-liquid interface, progressively growing downward over time. This phenomenon was consistently observed, demonstrating ultrasonic stimulation’s clear and reproducible effect on nucleation. The ultrasonic ice seeding method achieved a nucleation induction success rate exceeding 99% through repeated trials, highlighting its reliability and efficiency. These findings establish a solid foundation for subsequent hepatocyte cryopreservation experiments by providing a controlled and highly effective approach to ice nucleation.

### 3.4 Effect of ultrasonic intensities on hepatocyte cell cryopreservation

**FIGURE 6:**
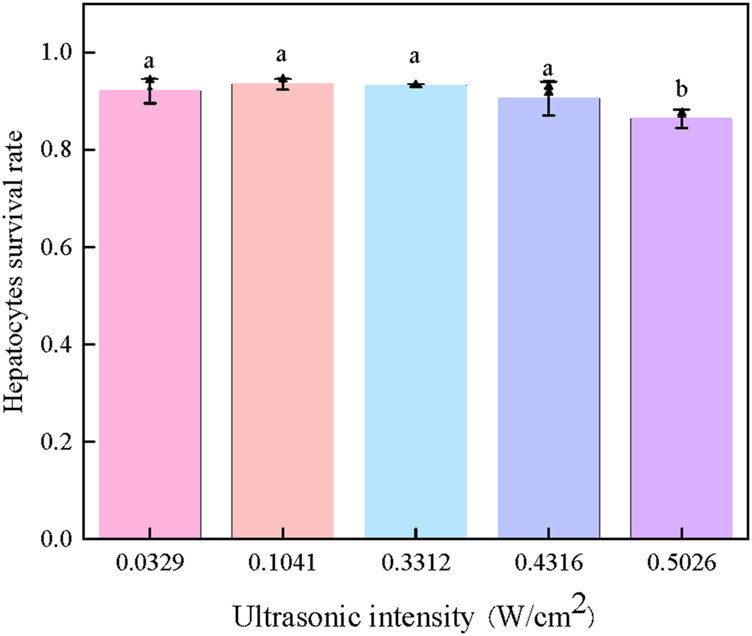
Effect of intensities of ultrasonic ice seeding on the survival rate of hepatocyte cells [Bars with a and b are significantly different (p < 0.05)].

To investigate the effect of ultrasonic intensity on hepatocyte cryopreservation, experiments were conducted under fixed conditions with 5% (v/v) Me_2_SO and an ice seeding temperature of −9°C (Figure 6).

As shown in Figure 6, when the ultrasonic intensity remained below 0.4316 W/cm^2^, the cell survival rate consistently exceeded 90%, indicating that low-intensity ultrasound had no apparent detrimental effects on cell viability. However, a significant decline in survival rate was observed when the ultrasonic intensity surpassed this threshold, suggesting potential ultrasound-induced cellular damage.

These findings highlight the existence of an optimal ultrasonic intensity range for effective ice seeding while maintaining high cell viability. When applied at an appropriate power level and duration, ultrasound can facilitate controlled nucleation without compromising cell integrity, allowing hepatocyte survival rates to exceed 90%. This result underscores the importance of fine-tuning ultrasonic parameters to maximize cryopreservation efficiency while minimizing potential adverse effects.

### 3.5 Effect of processing methods on cryopreservation of hepatocyte cells

**FIGURE 7:**
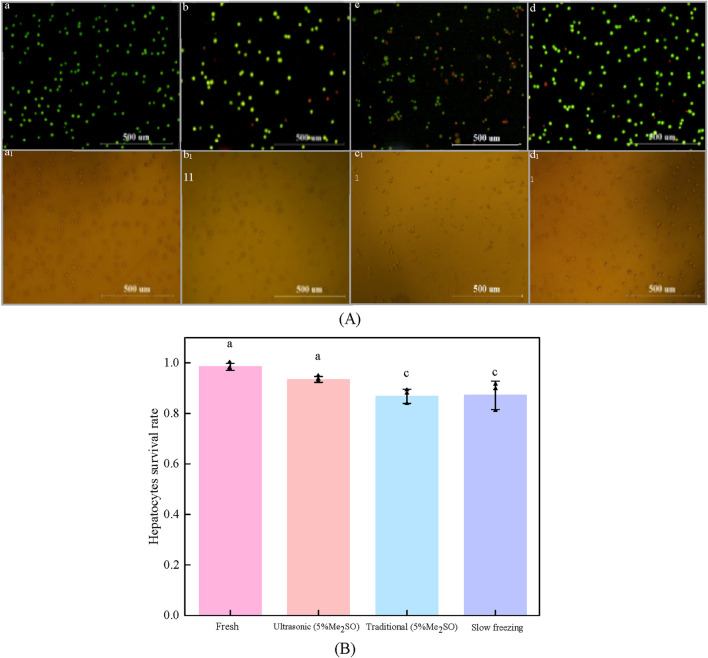
The effect of processing methods on cryopreservation of hepatocyte cells. **(A)** Fluorescence images of hepatocyte cells with different processing methods: **(a)** fresh hepatocyte cells; **(b)** Ultrasonic ice seeding method; **(c)** traditional ice seeding method; **(d)** Slow freezing method; **(a1)** ∼ **(d1)** Corresponding to the bright field images of **(a–d)** respectively; **(B)** Hepatocyte cells survival rate of different treatment methods [Bars with a and c are significantly different (p < 0.05)].

Figure 7 shows the effect of processing methods on cryopreservation of hepatocyte cells.

As shown in Figure 7, the volume concentration of Me_2_SO is 5%, and the pre-cooling temperature is −9°C. Compared with the traditional ice seeding method, the survival rate of ultrasonic ice seeding is greatly improved, and the hepatocyte cell survival rate reaches (93.4 ± 1.1) %, which is not significantly different from the fresh group. The hepatocyte survival rate of the traditional ice seeding method is only (86.71 ± 2.8) %. Ultrasonic ice seeding has more advantages than conventional ice seeding. The hepatocyte cell survival rate of the slow freezing method is (87.2 ± 5.6) %, which is not significantly different from the traditional ice seeding group but is significantly different from the ultrasonic ice seeding group.

The enhanced effectiveness of ultrasonic ice seeding may be attributed to its streamlined operation. The cryotube remains within the container throughout the process, ensuring consistent temperature control and minimizing fluctuations. In contrast, the traditional ice seeding method requires repeated removal of the cryotube, leading to significant temperature variations that may negatively impact cell survival. These findings demonstrate the advantages of ultrasonic ice seeding, including its high success rate, improved preservation efficiency, and superior cell viability compared to conventional methods.

### 3.6 Effect of treatment methods on hepatocyte cell morphology

**FIGURE 8:**
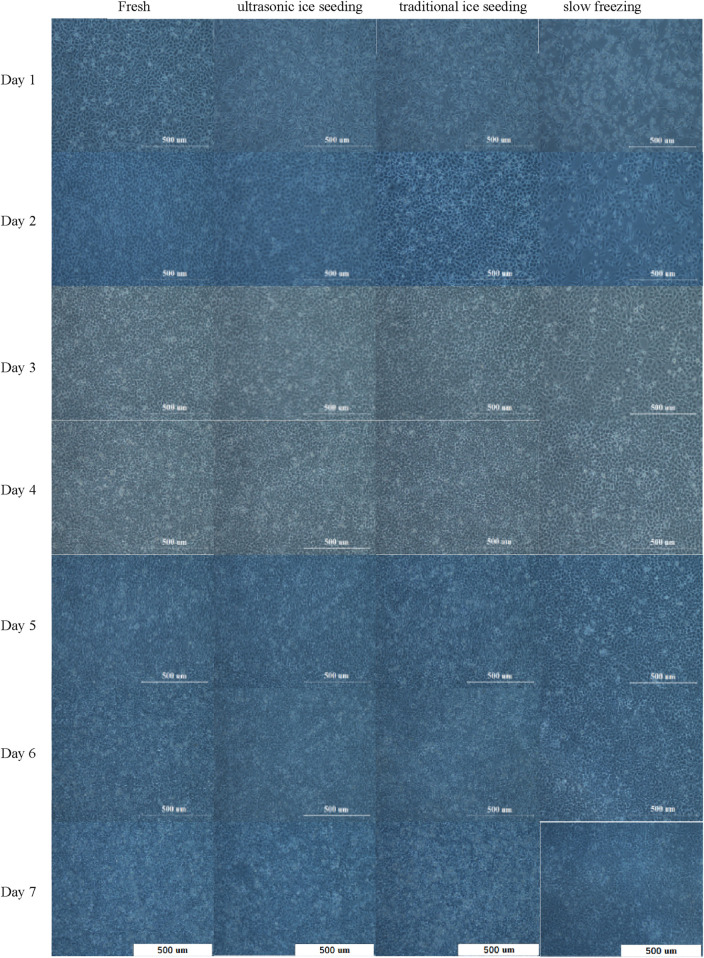
The hepatocyte cell morphology cultured for 7 days by different cryopreservation methods.

To explore the long-term effects of different cryopreservation methods on the metabolic activity of hepatocyte cells, we used three different treatment methods: ultrasonic ice seeding, traditional ice seeding, slow freezing, and fresh group as control. Ultrasound ice seeding, traditional ice seeding and slow-freezing hepatocyte cells developed a characteristic polygonal-shaped monolayer with typical binuclear cells on day 1, as shown in Figure 8.

The number of adherent hepatocyte cells in the fresh group is more than that in the ultrasonic and traditional ice-seeding groups. The slow-freezing group has the largest number of dead cells. Hepatocyte cell numbers began to increase on the first day. Clear and bright intercellular spaces could be seen on the second day. The cells grew wildly from the second to the sixth day, and the hepatocyte cells gradually became denser until no gaps existed. The number of dead hepatocyte cells in the four groups on the sixth day increased. On the seventh day, dead hepatocyte cells increased significantly and appeared in suspension because the hepatocyte cells were adherent. On the sixth day, the culture flask grew to a saturation density, and the hepatocyte cells could not continue to grow and reproduce. After 7 days of cell culture, we found that the hepatocyte cells in the fresh group grew the fastest, and the cells in the ultrasonic ice seeding group grew rapidly. There was no significant difference from the fresh group. The traditional ice seeding group’s cell growth rate is slower than that of the fresh and ultrasonic ice seeding groups. The slow-freezing group has slow hepatocyte cell growth and the largest number of dead cells. In short, deep cryopreservation has the most significant impact on preserving slow-frozen cells and the most negligible impact on preserving ultrasonic ice-planted cells.

### 3.7 Effects of treatment methods on hepatic function

#### 3.7.1 Urea production

Detoxification is a vital function of the liver. Ammonia is a highly toxic base produced during the deamination of amino acids. Hepatocyte cells almost exclusively metabolize ammonia into much more poisonous urea. As such, urea production is one of the most common markers of specific hepatic function. The supernatant was sampled to measure the urea production of hepatocyte cells, as shown in Table 3. The urea produced on the first and second days was between 1 and 1.5 mol/L. From the third to the fourth day, the urea secretion increased significantly, and the urea secretion reached the maximum on the sixth day. On the seventh day, urea production decreased. There was no significant difference between the fresh, ultrasonic, and traditional ice seeding groups on the first, fourth, fifth, and seventh days. Still, there was a significant difference from the slow-freezing group.

**TABLE 3:** Urea production, albumin synthesis, and glucose secretion of hepatocytes cultured for 7 days under different cryopreservation methods.

Secretion products	Time	Mean ± SD				P values					
		Fresh	Ultrasonic ice seeding	Traditional ice seeding	Slow freezing	Fresh-Ultr	Fresh-Tra	Fresh-Slow	Ultr -Tra	Ultr -Slow	Tra -Slow
Urea (mol/L)	Day 1	1.32 ± 0.04	1.32 ± 0.10	1.21 ± 0.04	1.09 ± 0.02	1.000	0.699	<0.05	0.703	<0.05	<0.05
	Day 2	1.38 ± 0.11	1.32 ± 0.15	1.26 ± 0.12	1.15 ± 0.04	0.919	0.618	0.136	0.924	0.314	0.617
	Day 3	3.44 ± 0.20	3.10 ± 0.26	2.93 ± 0.67	2.36 ± 0.23	0.732	0.442	<0.05	0.948	0.169	0.339
	Day 4	6.49 ± 0.10	6.38 ± 0.08	6.24 ± 0.16	5.17 ± 0.65	0.974	0.793	<0.05	0.955	<0.05	<0.05
	Day 5	7.14 ± 0.11	7.04 ± 0.05	7.01 ± 0.11	6.55 ± 0.19	0.796	0.637	<0.05	0.991	<0.05	<0.05
	Day 6	7.36 ± 0.10	7.24 ± 0.04	7.10 ± 0.09	6.38 ± 0.04	0.276	<0.05	<0.05	0.163	<0.05	<0.05
	Day 7	6.90 ± 0.23	6.72 ± 0.02	6.60 ± 0.23	5.92 ± 0.25	0.737	0.375	<0.05	0.899	<0.05	<0.05
Albumin (g/L)	Day 1	9.30 ± 0.01	9.45 ± 0.46	9.30 ± 0.01	8.37 ± 0.04	1.000	0.848	<0.05	0.856	<0.05	<0.05
	Day 2	10.85 ± 0.10	11.01 ± 0.13	10.85 ± 0.10	9.92 ± 0.06	<0.05	<0.05	<0.05	0.219	<0.05	<0.05
	Day 3	27.41 ± 0.28	27.88 ± 0.19	27.41 ± 0.28	25.43 ± 0.21	0.871	<0.05	<0.05	0.129	<0.05	<0.05
	Day 4	31.01 ± 0.32	33.95 ± 3.17	31.01 ± 0.32	30.87 ± 0.19	0.998	0.146	0.126	0.188	0.162	1.000
	Day 5	32.57 ± 0.45	35.96 ± 0.97	32.57 ± 0.45	33.01 ± 0.01	0.999	<0.05	<0.05	<0.05	<0.05	<0.05
	Day 6	35.86 ± 0.86	38.28 ± 0.16	35.86 ± 0.86	31.16 ± 0.27	0.180	<0.05	<0.05	<0.05	<0.05	<0.05
	Day 7	21.91 ± 0.14	24.82 ± 0.93	21.91 ± 0.14	20.05 ± 0.37	0.8	<0.05	<0.05	<0.05	<0.05	<0.05
Glucose (mol/L)	Day 1	5.61 ± 0.26	5.52 ± 0.27	4.33 ± 0.37	2.32 ± 0.41	0.833	<0.05	<0.05	<0.05	<0.05	<0.05
	Day 2	5.95 ± 0.26	5.82 ± 0.34	4.92 ± 0.35	3.33 ± 0.33	0.635	<0.05	<0.05	<0.05	<0.05	<0.05
	Day 3	6.33 ± 0.27	6.18 ± 0.44	5.28 ± 0.39	4.03 ± 0.39	<0.05	<0.05	<0.05	<0.05	<0.05	<0.05
	Day 4	7.59 ± 0.24	6.93 ± 0.28	6.85 ± 0.34	5.07 ± 0.36	<0.05	<0.05	<0.05	<0.05	<0.05	<0.05
	Day 5	8.10 ± 0.31	7.22 ± 0.36	7.18 ± 0.25	5.21 ± 0.40	<0.05	<0.05	<0.05	0.941	<0.05	<0.05
	Day 6	9.09 ± 0.40	8.21 ± 0.22	7.98 ± 0.35	5.85 ± 0.27	<0.05	<0.05	<0.05	<0.05	<0.05	<0.05
	Day 7	6.78 ± 0.14	6.21 ± 0.20	5.66 ± 0.32	3.89 ± 0.37	<0.05	<0.05	<0.05	<0.05	<0.05	<0.05

There was no significant difference between the fresh group, ultrasonic ice seeding, and traditional ice seeding on the second and third days. Still, there was a significant difference from slow freezing. On the sixth day, urea secretion reached its peak. There was no significant difference between ultrasonic ice seeding and the fresh group, and there were significant differences between traditional ice seeding and slow freezing.

These results indicate that ultrasonic ice seeding better preserves hepatocyte metabolic function than traditional ice seeding and slow freezing. The ability of hepatocytes in the ultrasonic ice seeding group to maintain near-native urea production levels suggests that this method offers a superior cryopreservation approach, minimizing cellular damage and preserving liver-specific functions more effectively.

#### 3.7.2 Albumin secretion

Albumin is the most abundant blood protein, almost entirely produced by the liver, so it is considered the most important sign of hepatocyte anabolism, as shown in Table 3.

From the first day to the sixth day, the albumin secretion increased with time, and it began to decrease after reaching its peak on the sixth day. On day 1, albumin levels were comparable among the fresh, ultrasonic ice seeding, and traditional ice seeding groups, while the slow-freezing group exhibited significantly lower secretion, suggesting that slow freezing may negatively impact hepatocyte function. By day 2, no significant difference was observed between the ultrasonic and traditional ice seeding groups. Still, both displayed significantly higher albumin secretion than the slow-freezing group, further highlighting the detrimental effects of slow-freezing. By day 3, there was no significant difference between the ultrasonic ice seeding and fresh groups. The ultrasonic and traditional ice seeding groups were not significantly different, and there were significant differences between the other groups. On day 4, there was no significant difference between the groups. From days 5 to 7, the ultrasonic ice seeding group consistently maintained albumin secretion levels comparable to the fresh group, while the traditional ice seeding and slow-freezing groups demonstrated significantly lower values. It suggests that ultrasonic ice seeding better preserves hepatocyte function over an extended culture period, ensuring sustained protein synthesis capacity.

#### 3.7.3 Glucose secretion

The liver plays a vital role in the process of glucose metabolism. Glucose secretion increases with time, peaks on the sixth day, and decreases on the seventh day in Table 3. Generally, the glucose content increases first and then decreases. This trend indicates an initial enhancement of metabolic activity, followed by a gradual decline, likely due to cellular density reaching saturation. On the first day, there was no significant difference in glucose secretion between the ultrasonic ice seeding group and the fresh group, and there were significant differences among the other groups. On the second day, there was no significant difference in glucose secretion between the ultrasonic ice seeding group and the fresh group, and there was no significant difference from the traditional ice seeding group. There were significant differences among the other groups. From the third day to the seventh day, there were significant differences among almost all groups, and the glucose secretion of the fresh group was higher than that of the ultrasonic ice seeding group. The ultrasonic ice seeding group is higher than the traditional ice seeding group. The traditional ice-seeding group is higher than the slow-freezing group.

## 4 Conclusion

The application of ultrasound-induced ice nucleation has been extensively explored in the cryopreservation of valuable food products, vitamins, and nutrients, as well as in the freeze concentration and lyophilization of pharmaceuticals and vaccines (Midya and Bandyopadhyay, 2024; Grover and Negi, 2023; Kazarin et al., 2023; Mokhova et al., 2023; Parale et al., 2024; Song et al., 2024). However, its use in the cryopreservation of cells remains largely unexplored. This study integrates ultrasound-mediated ice nucleation with liver cell cryopreservation to investigate its effects on cell viability and liver function.

In the study of ultrasound-assisted ice crystallization, ultrasound energy dissipation occurs due to heat loss during transmission and energy absorption by the cryovials containing the cells. To quantify this dissipation, the heat balance equation was employed to calculate the ultrasound power and intensity within the cell solution.

The ultrasound-induced ice nucleation system demonstrated a rapid and highly efficient nucleation capability, achieving a success rate exceeding 99%. Moreover, this technique significantly enhanced liver cell survival rates post-cryopreservation, outperforming traditional ice nucleation methods. Ultrasound-mediated ice nucleation effectively reduced supercooling in the cell solution, mitigating large temperature differentials caused by latent heat release and minimizing intracellular stress. This process ultimately facilitated a more controlled cooling rate, reducing intracellular ice formation.

Ultrasound propagates through liquids as alternating positive and negative pressure waves. The negative pressure phase disrupts the liquid’s structural integrity at sufficient power and frequency, leading to cavitation bubble formation. These bubbles grow over time as dissolved gases enter them and eventually collapse under positive pressure cycles (Cheng et al., 2015). This cavitation process enhances heat and mass transfer during freezing, reduces interfacial energy, and promotes ice nucleation (Inada et al., 2001; Hu et al., 2013). However, excessively high and low ultrasound intensities were detrimental to liver cell cryopreservation. Experimental results indicate that at ultrasound intensities below 0.4316 W/cm^2^, cell survival rates remained above 90%, making this approach suitable for biobanking applications. However, at intensities exceeding this threshold, cell viability declined significantly. Therefore, optimizing ultrasound parameters is crucial.

Over a seven-day culture period, ultrasound-induced ice nucleation had the least impact on liver function compared to traditional ice nucleation, while slow freezing exhibited the most detrimental effects. These findings suggest that ultrasound-mediated ice nucleation can better preserve metabolic activity and functional integrity over extended periods. However, while short-term benefits have been established, further studies evaluating long-term cryopreserved cells are necessary to fully validate the advantages of this technique under optimal ultrasound conditions.

This study provides experimental evidence supporting the application of ultrasound-induced ice nucleation in liver cell cryopreservation and offers new insights into optimizing cryopreservation strategies. Future research should explore the applicability of this method to a broader range of cell types, including embryonic stem cells, immune cells, and other medically significant cell types, to assess its potential in diverse biomedical applications. Additionally, further investigations into the underlying mechanisms of ultrasound-mediated ice nucleation and optimization of ultrasound parameters could facilitate the development of tailored cryopreservation protocols for different cell types. Through these efforts, a more comprehensive understanding of ultrasound-assisted cryopreservation can be achieved, providing a robust theoretical and experimental foundation for advancing cell preservation technologies.

## Data Availability

The original contributions presented in the study are included in the article/supplementary material, further inquiries can be directed to the corresponding author.
